# Drug Discovery Prospect from Untapped Species: Indications from Approved Natural Product Drugs

**DOI:** 10.1371/journal.pone.0039782

**Published:** 2012-07-11

**Authors:** Feng Zhu, Xiao Hua Ma, Chu Qin, Lin Tao, Xin Liu, Zhe Shi, Cun Long Zhang, Chun Yan Tan, Yu Zong Chen, Yu Yang Jiang

**Affiliations:** 1 The Key Laboratory of Chemical Biology, Guangdong Province, Graduate School at Shenzhen, Tsinghua University, Shenzhen, Guangdong, People’s Republic of China; 2 Innovative Drug Research Centre and College of Chemistry and Chemical Engineering, Chongqing University, Chongqing, People’s Republic of China; 3 Bioinformatics and Drug Design Group, Department of Pharmacy, and Center for Computational Science and Engineering, National University of Singapore, Singapore; 4 NUS Graduate School for Integrative Sciences and Engineering, Singapore; Universidade Federal do Rio de Janeiro, Brazil

## Abstract

Due to extensive bioprospecting efforts of the past and technology factors, there have been questions about drug discovery prospect from untapped species. We analyzed recent trends of approved drugs derived from previously untapped species, which show no sign of untapped drug-productive species being near extinction and suggest high probability of deriving new drugs from new species in existing drug-productive species families and clusters. Case histories of recently approved drugs reveal useful strategies for deriving new drugs from the scaffolds and pharmacophores of the natural product leads of these untapped species. New technologies such as cryptic gene-cluster exploration may generate novel natural products with highly anticipated potential impact on drug discovery.

## Introduction

High-throughput screening and combinatorial chemistry based drug discovery efforts have not led to the expected drug productivity, raising renewed interest in searching drugs from nature [1]_ENREF_2. Various species have been extensively searched in the past [2]–[4] with few percentage of the identified bioactive natural products carried forward to derive 939 approved drugs [2], [5] that are composed of limited number of molecular scaffolds [6]. Natural products have fallen out of favor partly because of technology shift [7], diminishing returns and high re-discovery rates [8], [9], and supply and screening problems [1], [10]. Although new technologies are expected to overcome some of these problems and enable significantly expanded bioprospecting efforts [1], [7], [11], [12], questions remain unanswered about the prospect of deriving new drugs from untapped species at acceptable return rates.

As part of the efforts in probing these questions, we have recently studied the distribution patterns of nature-derived drugs in phylogenetic trees and shown that drug-productive species tend to be clustered in specific regions of phylogenetic space (drug-productive clusters) with most drugs derived from existing drug-productive families (families that have yielded at least one approved drugs) [5]. Further clues to the prospect of deriving drugs from untapped species and the effective drug discovery strategies may be gained from the analysis of approved drugs derived from previously untapped species, particularly those approved in recent decades. In this work, we analyzed the species origins of nature-derived drugs approved in 1991–2010 with respect to those approved in previous decades (1961–1990) to find the exploration trends indicative of future bioprospecting prospect and likely sources of untapped new drug-productive species. We also tracked development histories of several classes of approved nature-derived drugs to reveal effective strategies for deriving new drugs from the bioactive natural products isolated from these species.

While the analysis of the approved drugs may provide useful clues to the drug discovery prospect of untapped species, some aspects of drug discovery prospect may not be fully captured because the approved drugs are different from discovered drugs by the additional commercial and technological considerations. For instance, the pharmaceutical industries have moved away from antibiotics partly because these are less lucrative than drugs for chronic conditions [13]. The exploration of microbial species has been limited by the cost and efficiency of cultivation technologies, with the majority of microbial organisms remain uncultured [14], [15]. Therefore, caution needs to be exercised in interpreting the results of our analysis, particularly with the possibility that new technologies that explore cryptic gene-clusters and networks [11], [12], [16], [17], inter-species crosstalk [11], [18], [19] and high-throughput fermentation [20] may provide significantly expanded molecular scaffolds for natural product drug discovery [15].

**Table 1:** Historic data of the numbers of nature-derived approved drugs from previously untapped and previous drug-productive species, and the numbers of drug-productive species during every five-year period from 1961 to 2010.

Period	Number of nature-derived drugs in period			Number of drug productive species in period		
	Total number of drugs in period	Number (percent)of drugs frompreviously untapped species	Number (percent) of drugs from previous drug productive species	Total number of species	Number (percent)of previouslyuntapped species	Number (percent) of pervious drug productive species
1961–1965	37	22 (59.5%)	15 (40.5%)	42	32 (76.2%)	10 (23.8%)
1966–1970	26	10 (38.5%)	16 (61.5%)	26	10 (38.5%)	16 (61.5%)
1971–1975	36	8 (22.2%)	28 (77.8%)	30	11 (36.7%)	19 (63.3%)
1976–1980	78	49 (62.8%)	29 (37.2%)	80	56 (70.0%)	24 (30.0%)
1981–1985	108	30 (27.8%)	78 (72.2%)	68	31 (45.6%)	37 (54.4%)
1986–1990	133	24 (18.0%)	109 (82.0%)	63	30 (47.6%)	33 (52.4%)
1991–1995	117	17 (14.5%)	100 (85.5%)	60	25 (41.7%)	35 (58.3%)
1996–2000	126	9 (7.1%)	117 (92.9%)	58	10 (17.2%)	48 (82.8%)
2001–2005	124	12 (9.7%)	112 (90.3%)	70	19 (27.1%)	51 (72.9%)
2006–2011	46	5 (10.9%)	41 (89.1%)	44	5 (11.4%)	39 (88.6%)

## Materials and Methods

A total of 939 nature-derived approved drugs have been found from Newman and Cragg’s seminal work [2] and our own literature search [5]. Their species origins have been identified from comprehensive literature search by using combinations of such keywords as drug name and alternative names, species, natural product and nature, and the search results have been confirmed based on such descriptions as “originates from”, “derived from”, “isolated from”, or “comes from” a species. The corresponding species families of the host species of these drugs are from the NCBI taxonomy database [21]. The approval dates of these drugs were from Newman and Cragg’s work [2] and Drugs@FDA on FDA website (http://www.accessdata.fda.gov/scripts/cder/drugsatfda/index.cfm).

**Table 2:** List of the new drug-productive species emerged in 1991–2010 together with the list of approved drugs derived from each species since the first drug approval, and the corresponding species families and their exploration status at the time of first drug approval.

New Drug ProductiveSpecies Emergedin 1991–2010 (Yearof FirstDrug Approval)	Approved Drugs Derived from the Species(Year of Approval, Target(s), Therapeutic Class,Category of Source)	Species Family (Yearof First DrugApproval)	Exploration Status of Species Family at the Time of First Drug Approval
**Period of 2001–2010**			
Halichondria okadai (2010)	Eribulin (2010, Tubulin, anticancer, ND)	Halichondriidae (2010)	Newly emerged drug productive family inside existing drug productive cluster
Isaria sinclairii (2010)	Fingolimod (2010, Sphingosine-1-phosphate receptor S1PR1, immunosuppression, ND)	Cordycipitaceae (2010)	Newly emerged drug productive family inside existing drug productive cluster
Emericella rugulosa (2007)	Anidulafungin (2007, (1->3)beta-D-glucan synthase, antifungal, ND)	Trichocomaceae (1942)	Existing drug productive family
Sorangium cellulosum (2007)	Ixabepilone (2007, Tubulin, anticancer, ND)	Polyangiaceae (2007)	Newly emerged drug productive family inside existing drug productive cluster
Clitopilus scyphoides (2007)	Retapamulin (2007, Bacterial ribosome,antibacterial, ND)	Entolomataceae (2007)	Newly emerged drug productive family inside existing drug productive cluster
Ecteinascidia turbinate (2007)	Trabectedin (2007, DNA, anticancer, N)	Perophoridae (2007)	Newly emerged drug productive family inside existing drug productive cluster
Heloderma suspectum (2005)	Exenatide (2005, GLP1R, antidiabetic, ND)	Helodermatidae (2005)	Newly emerged drug productive family inside existing drug productive cluster
Aspergillus fumigates (2005)	Fumagillin (2005, METAP2, antiparasitic, N)	Trichocomaceae (1942)	Existing drug productive family
Conus geographus (2005)	Omega-conotoxin MVIIA (2005, Voltage-dependentN-type calcium channel, chronic pain relief, N)	Conidae (2005)	Newly emerged drug productive family inside existing drug productive cluster
Conus magus (2005)	Omega-conotoxin MVIIA (2005, Voltage-dependentN-type calcium channel, chronic pain relief, N)	Conidae (2005)	Newly emerged drug productive family inside existing drug productive cluster
Streptomyces filamentosus(2003)	Daptomycin (2003, Bacterial cell membrane,antibacterial, N)	Streptomycetaceae (1946)	Existing drug productive family
Galanthus elwesii (2002)	Galantamine (2002, Acetylcholinesterase, Alzheimer’s disease, N)	Amaryllidaceae (1983)	Existing drug productive family
Galanthus nivalis (2002)	Galantamine (2002, Acetylcholinesterase, Alzheimer’s disease, N)	Amaryllidaceae (1983)	Existing drug productive family
Galanthus woronowii (2002)	Galantamine (2002, Acetylcholinesterase, Alzheimer’s disease, N)	Amaryllidaceae (1983)	Existing drug productive family
Leucojum aestivum (2002)	Galantamine (2002, Acetylcholinesterase, Alzheimer’s disease, N)	Amaryllidaceae (1983)	Existing drug productive family
Lycoris radiate (2002)	Galantamine (2002, Acetylcholinesterase, Alzheimer’s disease, N)	Amaryllidaceae (1983)	Existing drug productive family
Lycoris squamigera (2002)	Galantamine (2002, Acetylcholinesterase, Alzheimer’s disease, N)	Amaryllidaceae (1983)	Existing drug productive family
Narcissus pseudonarcissus(2002)	Galantamine (2002, Acetylcholinesterase, Alzheimer’s disease, N)	Amaryllidaceae (1983)	Existing drug productive family
Cocos nucifera (2002)	Gefitinib (2002, EGFR, anticancer, S/NM), Erlotinib (2004, EGFR, anticancer, S/NM), Sunitinib (2006, VEGFR, anticancer, S/NM), Lapatinib (2007, EGFR and HER2, anticancer, S/NM), Pazopanib (2009, VEGFR, anticancer, S/NM)	Arecaceae (1978)	Existing drug productive family
Spinacia oleracea (2002)	Gefitinib (2002, EGFR, anticancer, S/NM), Erlotinib(2004, EGFR, anticancer, S/NM), Sunitinib (2006,VEGFR, anticancer, S/NM), Lapatinib (2007, EGFRand HER2, anticancer, S/NM), Pazopanib (2009,VEGFR, anticancer, S/NM)	Amaranthaceae (2002)	Newly emerged drug productive family outside existing drug productive cluster
Zea mays (2002)	Gefitinib (2002, EGFR, anticancer, S/NM), Erlotinib(2004, EGFR, anticancer, S/NM), Sunitinib (2006,VEGFR, anticancer, S/NM), Lapatinib (2007, EGFRand HER2, anticancer, S/NM), Pazopanib (2009,VEGFR, anticancer, S/NM)	Poaceae (1984)	Existing drug productive family
Coleophoma empetri (2002)	Micafungin (2002, Beta-(1,3)-Glucan synthase,antifungal, ND)	Coleophoma (2002)	Newly emerged drug productive family outside existing drug productive cluster
Brevibacillus laterosporus(2001)	Gusperimus (2001, Interleukin-2,immunosuppressant, ND)	Paenibacillaceae (1942)	Existing drug productive family
Lentzea albida (2001)	Imatinib (2001, Abl, anticancer, ND), Nilotinib (2007, Abl, anticancer, ND)	Actinosynnemataceae (1971)	Existing drug productive family
**Period of 1991–2000**			
Plectranthus barbatus (1999)	Colforsin daropate (1999, Adenylate cyclase, cardiotonic, ND)	Lamiaceae (1978)	Existing drug productive family
Corynebacterium diphtheria (1999)	Denileukin diftitox (1999, IL-2R, anticancer, B)	Corynebacteriaceae (1999)	Newly emerged drug productive family inside existing drug productive cluster
Lendenfeldia chondrodes (1998)	Miglitol (1998, Alpha-glucosidase, antidiabetic, ND), Miglustat (2003, Glucosylceramide synthase, anti-gaucher’s disease, ND)	Thorectidae (1998)	Newly emerged drug productive family inside existing drug productive cluster
Morus alba (1998)	Miglitol (1998, Alpha-glucosidase, antidiabetic, ND), Miglustat (2003, Glucosylceramide synthase, anti-gaucher’s disease, ND)	Moraceae (1998)	Newly emerged drug productive family inside existing drug productive cluster
Streptomyces toxytricini (1998)	Orlistat (1998, Lipase, antiobesity, ND)	Streptomycetaceae (1946)	Existing drug productive family
Citrus bergamia (1997)	Hesperetin (1997, Diglyceride acyltransferase, anti-cardiovascular disease, N)	Rutaceae (1900)	Existing drug productive family
Citrus limon (1997)	Hesperetin (1997, Diglyceride acyltransferase, anti-cardiovascular disease, N)	Rutaceae (1900)	Existing drug productive family
Haementeria officinalis (1997)	Lepirudin (1997, Thrombin, antithrombotic, ND), Bivalirudin (2000, Thrombin, anti-cardiovascular disease, ND), Hirulog (2000, Thrombin, anticoagulation, ND), Desirudin (2003, Thrombin, deep vein thrombosis, ND)	Glossiphoniidae (1997)	Newly emerged drug productive family inside existing drug productive cluster
Hirudo medicinalis (1997)	Lepirudin (1997, Thrombin, antithrombotic, ND), Bivalirudin (2000, Thrombin, anti-cardiovascular disease, ND), Hirulog (2000, Thrombin, anticoagulation, ND), Desirudin (2003, Thrombin, deep vein thrombosis, ND)	Hirudinidae (1997)	Newly emerged drug productive family inside existing drug productive cluster
Artemisia dracunculus (1996)	Latanoprost (1996, PGF receptor, antiglaucoma, ND), Bimatoprost (2001, PGF receptor, antiglaucoma, ND), Travoprost (2001, PGF receptor, antiglaucoma, ND)	Asteraceae (1975)	Existing drug productive family
Mus musculus (1995)	Edrecolomab (1995, EpCAM, anticancer, B), Daclizumab (1997, IL-2R, immunosuppressant, B), Palivizumab (1998, Fusion glycoprotein, antiviral, B), Trastuzumab (1998, HER-2, anticancer, B), Alemtuzumab (2001, CD52, anticancer, B), Efalizumab (2003, CD11a subunit of lymphocyte function-associated antigen 1, antipsoriatic, B), Omalizumab (2003, Human immunoglobulin E, antiasthmatic, B), Bevacizumab (2004, VEGF-A, anticancer, B), Natalizumab (2004, Alpha-4 integrin, anti-inflammatory, B), Tocilizumab (2005, IL-6R, immunosuppressant, B), Nimotuzumab (2006, EGFR, anticancer, B), Ranibizumab (2006, VEGF-A, anti-angiogenic, B)	Muridae (1994)	Newly emerged drug productive family inside existing drug productive cluster
Aspergillus unilateralis (1995)	Mycophenolate mofetil (1995, IMPDH2, immunosuppressant, ND), Mycophenolic acid (2003, IMPDH2, immunosuppressant, N)	Trichocomaceae (1942)	Existing drug productive family
Byssochlamys nivea (1995)	Mycophenolate mofetil (1995, IMPDH2, immunosuppressant, ND), Mycophenolic acid (2003, IMPDH2, immunosuppressant, N)	Trichocomaceae (1942)	Existing drug productive family
Penicillium bialowiezense (1995)	Mycophenolate mofetil (1995, IMPDH2, immunosuppressant, ND), Mycophenolic acid (2003, IMPDH2, immunosuppressant, N)	Trichocomaceae (1942)	Existing drug productive family
Penicillium carneum (1995)	Mycophenolate mofetil (1995, IMPDH2, immunosuppressant, ND), Mycophenolic acid (2003, IMPDH2, immunosuppressant, N)	Trichocomaceae (1942)	Existing drug productive family
Penicillium fagi (1995)	Mycophenolate mofetil (1995, IMPDH2, immunosuppressant, ND), Mycophenolic acid (2003, IMPDH2, immunosuppressant, N)	Trichocomaceae (1942)	Existing drug productive family
Penicillium roqueforti (1995)	Mycophenolate mofetil (1995, IMPDH2, immunosuppressant, ND), Mycophenolic acid (2003, IMPDH2, immunosuppressant, N)	Trichocomaceae (1942)	Existing drug productive family
Streptomyces argenteolus subsp. Toyonakensis (1995)	Saquinavir mesylate (1995, HIV-1 protease, anti-virus, S*/NM), Indinavir sulfate (1996, HIV-1 protease, anti-virus, S*/NM), Ritonavir (1996, HIV-1 protease, anti-virus, S*/NM), Neflinavir mesylate (1997, HIV-1 protease, anti-virus, S*/NM), Amprenavir (1999, HIV-1 protease, anti-virus, S*/NM), Lopinavir (2000, HIV-1 protease, anti-virus, S*/NM)	Streptomycetaceae (1946)	Existing drug productive family
Streptomyces testaceus (1995)	Saquinavir mesylate (1995, HIV-1 protease, anti-virus, S*/NM), Indinavir sulfate (1996, HIV-1 protease, anti-virus, S*/NM), Ritonavir (1996, HIV-1 protease, anti-virus, S*/NM), Neflinavir mesylate (1997, HIV-1 protease, anti-virus, S*/NM), Amprenavir (1999, HIV-1 protease, anti-virus, S*/NM), Lopinavir (2000, HIV-1 protease, anti-virus, S*/NM)	Streptomycetaceae (1946)	Existing drug productive family
Camptotheca acuminate (1994)	Irinotecan (1994, DNA topoisomerase I, anticancer, ND), Topotecan (1996, DNA topoisomerase I, anticancer, ND)	Cornaceae (1994)	Newly emerged drug productive family outside existing drug productive cluster
Mappia foetida (1994)	Irinotecan (1994, DNA topoisomerase I, anticancer, ND), Topotecan (1996, DNA topoisomerase I, anticancer, ND), Belotecan (2004, DNA topoisomerase I, anticancer, ND), Sphingosomal topotecan (2007, DNA topoisomerase I, anticancer, ND)	Icacinaceae (1994)	Newly emerged drug productive family inside existing drug productive cluster
Ophiorrhiza pumila (1994)	Irinotecan (1994, DNA topoisomerase I, anticancer, ND), Topotecan (1996, DNA topoisomerase I, anticancer, ND), Belotecan (2004, DNA topoisomerase I, anticancer, ND), Sphingosomal topotecan (2007, DNA topoisomerase I, anticancer, ND)	Rubiaceae (1940)	Existing drug productive family
Rattus norvegicus (1994)	Losartan potassium (1994, AGTR1, antihypertensive, S/NM), Valsartan (1996, AGTR1, antihypertensive, S/NM), Candesartan cilexetil (1997, AGTR2, antihypertensive, S/NM), Eprosartan (1997, AGTR1, antihypertensive, S/NM), Irbesartan (1997, AGTR1, antihypertensive, S/NM), Telmisartan (1999, AGTR1, antihypertensive, S/NM), Olmesartan medoxil (2002, Angiotensin II receptor 1, antihypertensive, S/NM)	Muridae (1994)	Newly emerged drug productive family inside existing drug productive cluster
Erwinia chrysanthemi (1994)	Pegaspargase (1994, L-asparaginase, anticancer, B)	Enterobacteriaceae (1969)	Existing drug productive family
Streptomyces hygroscopicus (1994)	Voglibose (1994, Alpha-glucosidase, antidiabetic, N), Zotarolimus (1996, mTOR, anticancer, ND), Sirolimus (1999, mTOR, immunosuppressive, N), Pimecrolimus (2001, Calcineurin, immunomodulating, ND), Vorinostat (2006, HDAC, anticancer, ND), Temsirolimus (2007, mTOR, anticancer, ND), Everolimus (2009, mTOR, anticancer, ND)	Streptomycetaceae (1946)	Existing drug productive family
Corylus avellana (1993)	Paclitaxel (1993, Tubulin, anticancer, N), Cabazitaxel (2010, Tubulin, anticancer, ND)	Betulaceae (1993)	Newly emerged drug productive family inside existing drug productive cluster
Seimatoantlerium tepuiense (1993)	Paclitaxel (1993, Tubulin, anticancer, N), Cabazitaxel (2010, Tubulin, anticancer, ND)	Amphisphaeriaceae (1993)	Newly emerged drug productive family outside existing drug productive cluster
Taxus baccata (1993)	Paclitaxel (1993, Tubulin, anticancer, N), Docetaxel (1995, Tubulin, anticancer, ND), Cabazitaxel (2010, Tubulin, anticancer, ND)	Taxaceae (1993)	Newly emerged drug productive family inside existing drug productive cluster
Taxus brevifolia (1993)	Paclitaxel (1993, Tubulin, anticancer, N), Docetaxel (1995, Tubulin, anticancer, ND), Cabazitaxel (2010, Tubulin, anticancer, ND)	Taxaceae (1993)	Newly emerged drug productive family inside existing drug productive cluster
Taxus wallichiana (1993)	Paclitaxel (1993, Tubulin, anticancer, N), Docetaxel (1995, Tubulin, anticancer, ND), Cabazitaxel (2010, Tubulin, anticancer, ND)	Taxaceae (1993)	Newly emerged drug productive family inside existing drug productive cluster
Streptomyces tsukubaensis (1993)	Tacrolimus (1993, Calcineurin, immunosuppressant, N)	Streptomycetaceae (1946)	Existing drug productive family
Larrea tridentate (1992)	Masoprocol (1992, 5-LOX, anticancer, N)	Zygophyllaceae (1978)	Existing drug productive family
Agaricus bisporus (1991)	Calcipotriol (1991, Vitamin D receptor, antipsoriatic, ND), Tacalcitol (1993, Vitamin D receptor, antipsoriatic, ND), Paricalcitol (1998, Vitamin D receptor, calcium metabolism, ND), Doxercalciferol (1999, Vitamin D receptor, calcium metabolism, ND)	Agaricaceae (1991)	Newly emerged drug productive family inside existing drug productive cluster
Leontice leontopetalum (1991)	Doxacurium chloride (1991, CHRNA2, muscle relaxant, S*), Mivacurium chloride (1992, CHRNA2, muscle relaxant, S*), Cisatracurium besilate (1995, nAChR, muscle relaxant, S*)	Berberidaceae (1978)	Existing drug productive family
Callistemon citrinus (1991)	Nitisinone (1991, 4HPPD, antityrosinaemia, ND)	Myrtaceae (1991)	Newly emerged drug productive family inside existing drug productive cluster

The categories of drug source are based on the definition of Newman and Cragg [2]: “B” Biological; usually a large (>45 residues) peptide or protein either isolated from an organism/cell line or produced by biotechnological means in a surrogate host,“N” Natural product, “ND” Derived from a natural product and is usually a semisynthetic modification, “S*” Made by total synthesis, but the pharmacophore is/was from a natural product.“V” Vaccine, “NM” Natural product mimic.

## Results and Discussion

**Table 1** presents the statistics of drugs approved in every five-year period of 1961–2010 (divided into drugs derived from previously untapped and previous drug-productive species respectively), and the statistics of drug-productive species that have produced drugs in each period. There are 46–126 nature-derived drugs approved in every 5-year periods since 1991, 7.1%–14.5% of which are from previously untapped species (i.e. untapped before the specific 5-year period) and these species represent 11.4%–41.7% of the drug-productive species that have yielded approved drugs in 1991–2010. In contrast, there are 26–133 nature-derived drugs in every 5-year period of 1961–1990, 18.0%–62.8% of which are from previously untapped species and these species represent 36.7%–76.2% of the drug-productive species in 1961–1990. While the percentages have been reduced to some extent, the recent trends of substantial percentage of drugs and substantial percentage of drug-productive species being from previously untapped species strongly suggests that the untapped drug-productive species is unlikely near extinction, and future bioprospecting efforts are expected to yield new drugs at comparable levels. This is consistent with an earlier analysis of the largest antibiotic-producing genus *Streptomyces* which suggests that the new compound discovery rate from the unexplored strains of *Streptomyces* would not decline for several decades and 15–20 antibiotics would be discovered each year at the 1995 exploration level [9]. It is also consistent with the estimated high antibiotic production frequencies by the untapped strains of the *actinomycetes* class (5×10^−6^–2×10^−1^ in screening 10^4^–10^7^ strains) [22].

**Figure 1:**
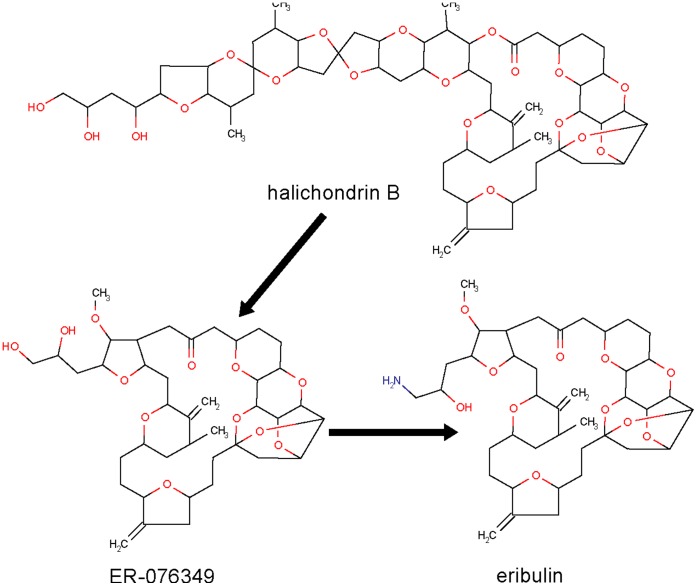
The route of deriving eribulin (approved in 2009) from natural product halichondrin B.

**Figure 2:**
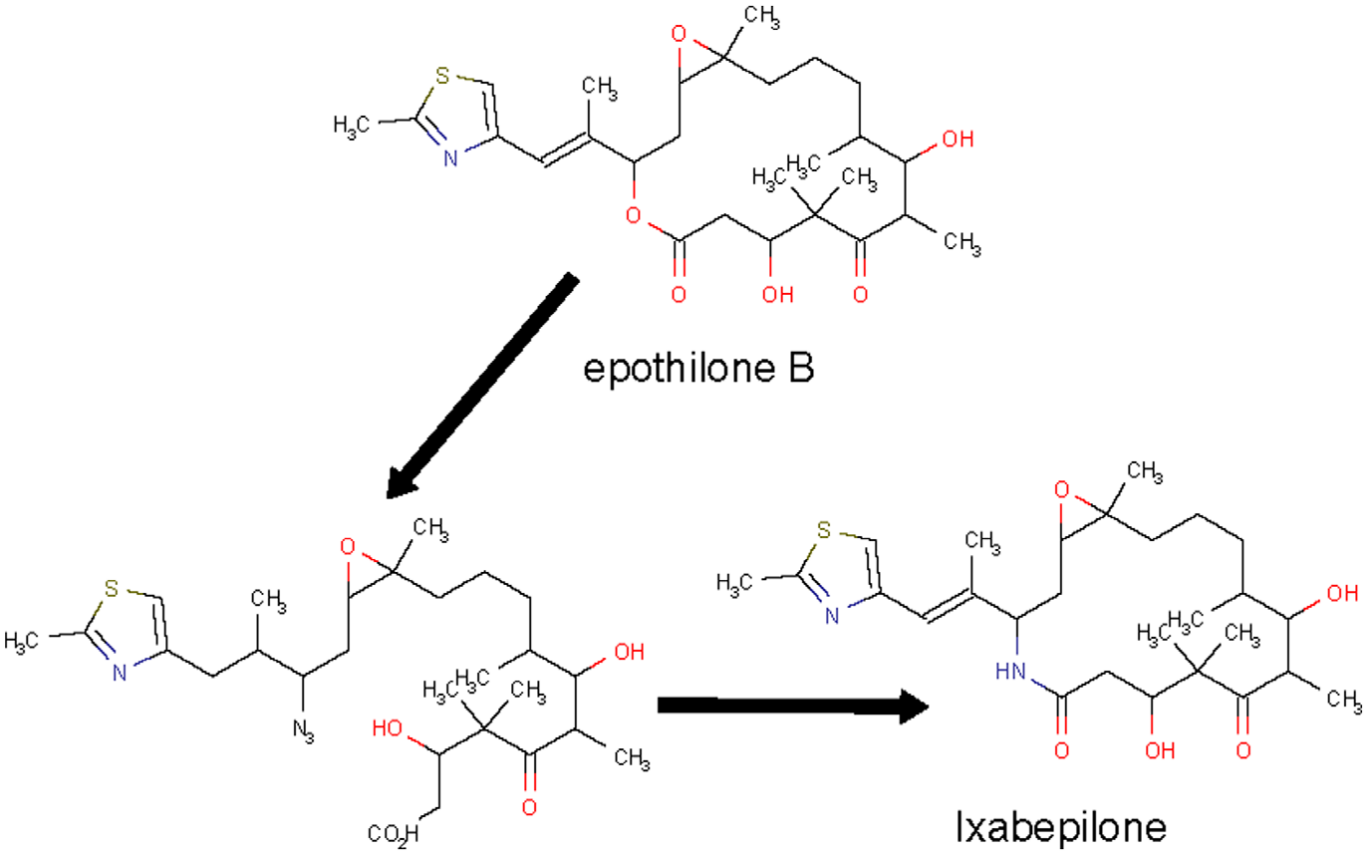
The route of deriving Ixabepilone (approved in 2009) from natural product epothilone B.

**Figure 3:**
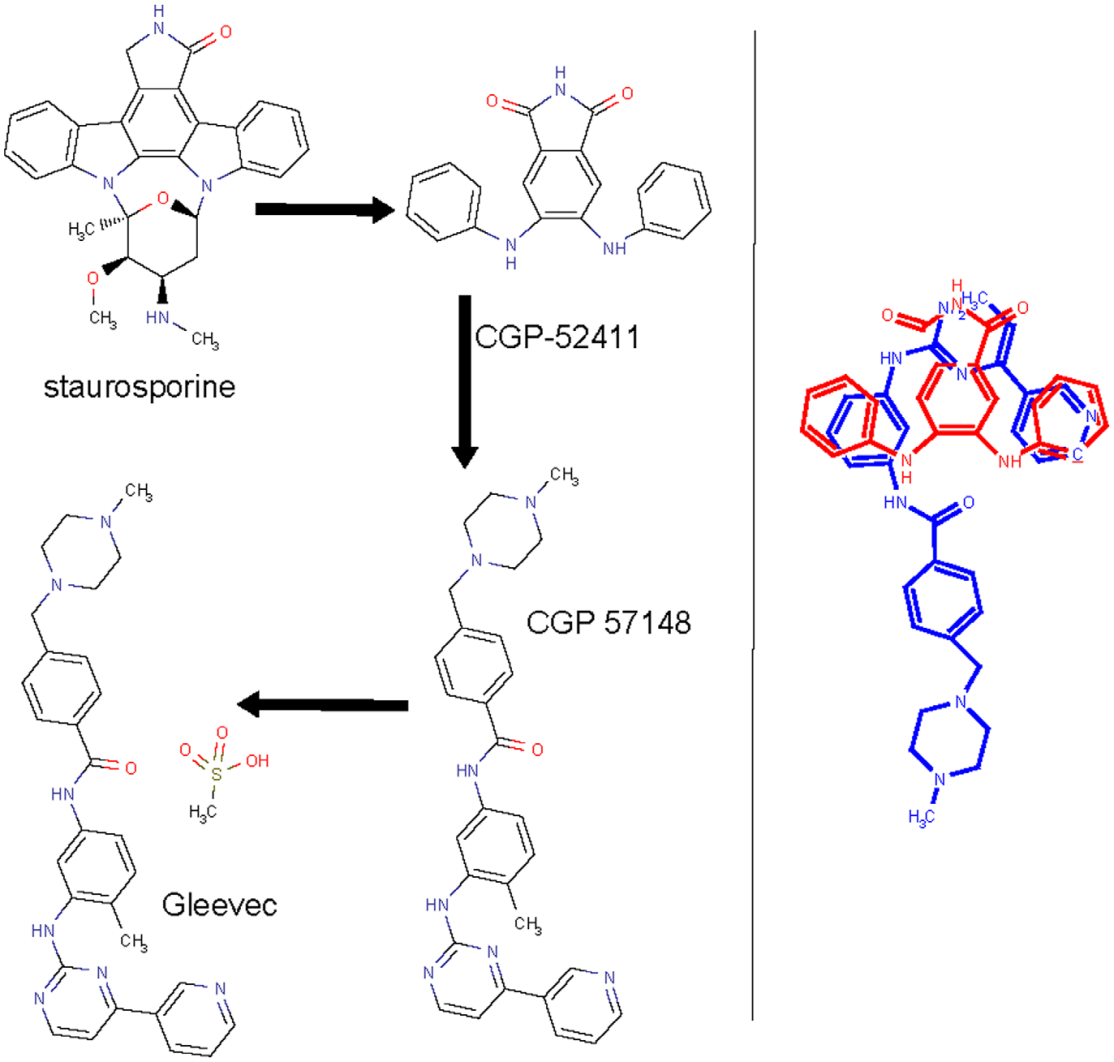
The route of deriving Gleevec (approved in 2001) from natural product staurosporine. The right hand side shows the pharmacophoric supposition of CGP 57148 and CGP-52411 [37].

**Table 2** provides the list of the new drug-productive species emerged in 1991–2010 together with the approved drugs derived from these species since the first drug approval. There are 59 new drug-productive species emerged in this period, 33 (55%) of which are from existing drug-productive species families and another 22 (37%) of which are from new species families in existing drug-productive clusters. These suggest a high probability of finding new drug-productive species from existing drug-productive families or new families located in existing drug-productive clusters. This coincides with the reported refocused efforts for finding new antibiotics from new sources in the *actinomycetes* class and *cyanobacteria* phylum [22], which cover an existing drug-productive cluster (*Actinomycetales* cluster) and an existing drug-productive family (*Symploca* family) respectively [5].

From the time of their first drug approval to the end of 2010, these 59 new drug-productive species have yielded 85 approved drugs, 12 (14.1%) and 14 (16.5%) of which are unmodified natural products and biologics respectively, while the majority (69.4%) of the 59 drugs have been derived from bioactive natural products by semi-synthetic modification, structural mimicking or pharmacophore mapping to overcome problems frequently encountered by bioactive natural products such as weak potency [23], low target selectivity [24], toxicity [25], undesired pharmacokinetic properties [26], and supply issues [27]. Retrospective study of the case histories of several classes of recently approved drugs reveal useful strategies for deriving new drugs from bioactive natural products from untapped species to overcome these frequently encountered problems.

Since the discovery of Paclitaxel [28], extensive efforts have been directed at the search of tubulin interacting anticancer drugs from species other than the tapped taxus species, leading to the identification of a number of natural products from untapped species [26], [27]. Two compounds, homohalichondrin B and halichondrin B, from previously untapped western Pacific sponge *Halichondria* and *Axinella* have shown strong cytotoxic and tubulin polymerization inhibitory activities, but faced with supply problems [27], Subsequent works in deriving simplified and synthetically accessible agents based on the halichondrin B skeleton have led to the discovery of eribulin approved in 2009 [27] (**Figure 1**). Further efforts have also led to the identification of epothilones, particularly epothilone B, from the previously untapped myxobacterium Sorangium cellulosum species as tubulin interacting anticancer agents with potent cytotoxic activity toward paclitaxel-sensitive and paclitaxel-resistant cells [26]. But these compounds are prone to the inactivation by esterase cleavage, and in an effort of overcoming this problem semisynthetic lactam analogs of epothilone B has been derived which led to the discovery of Ixabepilone approved in 2009 [26] (**Figure 2**).

The identification of BCR-ABL as a key target of chronic myeloid leukemia has prompted extensive efforts in identifying anticancer ABL inhibitor drugs [29]. Design works based on the pharmacophore of a nonselective pan-kinase inhibitor staurosporine from the previously untapped *Lentzea albida* species have resulted in an EGFR selective inhibitor CGP 52411 [24] and subsequently a potent ABL selective inhibitor CGP 57148 [30]. CGP 57148 has undesired pharmacokinetic properties, which is improved by formulating it with mesylate salt leading to Gleevec, a milestone anticancer kinase inhibitor approved in 2001 [31] (**Figure 3**).

The identification of HIV-1 protease as a key anti-HIV target has also motivated extensive efforts in identifying HIV-protease inhibitor drugs. Before the start of HIV-1 protease inhibitor projects, pepstatin from previously untapped *Streptomyces argenteolus subsp. Toyonakensis* and *Streptomyces testaceus* species has been found to show weak human rennin inhibitory activities, and more potent peptidomimetic human rennin inhibitors have been derived from it by further optimizing binding configuration to better mimicking substrate binding [23]. Based on these works, a series of potent peptidomimetic HIV-protease inhibitors have been designed [32], [33], leading to a series of approved anti-HIV drugs Saquinavir, Indinavir, Ritonavir, Neflinavir, Amprenavir, and Lopinavir in 1995–2000.

In 2001–2011, some new molecular scaffolds have been derived from both previously explored species and previously untapped species. For instance, the 4-anilinoquinazoline scaffold in Gefitinib, Erlotinib and Lapatinib has been derived based on the pharmacophore of a selective kinase inhibitor olomoucine, a semi-synthetic derivative of zeatin from Maize, *Cocos nucifera, Spinacia oleracea*, and *Pisum sativum* species that have been previously explored for deriving cardiovascular and anthelmintic agents [34]–[36]. The 2-phenylaminopyrimidine scaffold in Imatinib and Nilotinib has been derived based on the pharmacophore of a nonselective pan-kinase inhibitor staurosporine from the previously untapped *Lentzea albida* species [24], [30], [31]. During the same period of time, many of the existing molecular scaffolds (those found in nature-derived drugs approved before the period) continue to contribute new approved drugs. Examples are the tetracycline scaffold of Minocycline, Methacycline and Tigecycline approved in 2001 and 2005 respectively, and the steroide scaffold of Falecalcitrol and Acetyldigitoxin approved in 2001 and 2002 respectively.

While most of the 2001–2011 approved nature-derived drugs targeting previously-explored pathways, some previously un-targeted pathways have become highly successfully targeted. These are MAPK, ErbB, mTOR, Brc-Abl regulated, and hematopoietic cell lineage pathways targeted by 7, 6, 3, 3 and 3 new drugs respectively. These pathways become successfully targeted partly because of the derivation of selective kinase inhibitory scaffolds based on the pharmacophores of zeatin derivatives [34]–[36] and staurosporine [24], [30], [31] etc. Some previously successfully explored pathways continue to be successfully targeted. Specifically, the calcium signaling, insulin signaling, and Toll-like receptor pathways are targeted by 10, 4, and 6 drugs approved before 2001 and 5, 4, and 4 drugs approved during 2001–2011 respectively.

Our analysis suggests that untapped drug-productive species are unlikely to be near extinction, and there is a high probability for finding new drug-productive species in the existing drug-productive families and drug-productive clusters. New technologies that explore cryptic gene-clusters [11], pathways [12], [17], inter-species crosstalk [11], [18], [19] and high-throughput fermentation [20] enable the generation of significantly more diverse groups of novel natural products, which has been anticipated to have some impact on drug productivity from nature. Some of the revolutionary new drugs of novel targets, such as Gleevec and Gefitinib approved in recent years and Saquinavir and Paclitaxel from earlier years, as well as new drugs of existing targets, have been derived from bioactive natural products of previously untapped species. Retrospective analysis of the case histories of these drugs reveals useful strategies for deriving new drugs from these natural sources.
